# Conversational Agents in the Treatment of Mental Health Problems: Mixed-Method Systematic Review

**DOI:** 10.2196/14166

**Published:** 2019-10-18

**Authors:** Hannah Gaffney, Warren Mansell, Sara Tai

**Affiliations:** 1 Division of Psychology & Mental Health School of Health Sciences, Faculty of Biology, Medicine and Health University of Manchester Manchester United Kingdom

**Keywords:** artificial intelligence, mental health, stress, pychological, psychiatry, therapy, computer-assisted, conversational agent, chatbot, digital health

## Abstract

**Background:**

The use of conversational agent interventions (including chatbots and robots) in mental health is growing at a fast pace. Recent existing reviews have focused exclusively on a subset of embodied conversational agent interventions despite other modalities aiming to achieve the common goal of improved mental health.

**Objective:**

This study aimed to review the use of conversational agent interventions in the treatment of mental health problems.

**Methods:**

We performed a systematic search using relevant databases (MEDLINE, EMBASE, PsycINFO, Web of Science, and Cochrane library). Studies that reported on an autonomous conversational agent that simulated conversation and reported on a mental health outcome were included.

**Results:**

A total of 13 studies were included in the review. Among them, 4 full-scale randomized controlled trials (RCTs) were included. The rest were feasibility, pilot RCTs and quasi-experimental studies. Interventions were diverse in design and targeted a range of mental health problems using a wide variety of therapeutic orientations. All included studies reported reductions in psychological distress postintervention. Furthermore, 5 controlled studies demonstrated significant reductions in psychological distress compared with inactive control groups. In addition, 3 controlled studies comparing interventions with active control groups failed to demonstrate superior effects. Broader utility in promoting well-being in nonclinical populations was unclear.

**Conclusions:**

The efficacy and acceptability of conversational agent interventions for mental health problems are promising. However, a more robust experimental design is required to demonstrate efficacy and efficiency. A focus on streamlining interventions, demonstrating equivalence to other treatment modalities, and elucidating mechanisms of action has the potential to increase acceptance by users and clinicians and maximize reach.

## Introduction

### Rationale

Conversational agents are software programs that use artificial intelligence to simulate a conversation with a user through written text or voice. Recent everyday examples include digital assistants such as Siri (Apple), Cortana (Microsoft), Google Now, and Alexa (Amazon) [1]. The first conversational agent of this kind was ELIZA [2], which was programmed to mimic conversation with a Rogerian psychotherapist using typed text. In the 50 years since ELIZA, interest in conversational agents and artificial intelligence has waxed and waned, and this is reflected in publication rates over time [3]. However, significant advances in technology over the past 2 decades have facilitated the design of conversational agents that can undertake evermore complex tasks [4]. This has resulted in an explosion of publications in this area, particularly since 2009 [3].

Evidence has begun to accumulate around the potential benefits of conversational agents in diverse fields [5] within health and medical care [6] and specifically in mental health [7-11]. Increased access to information through the internet and mobile phones has highlighted the potential for conversational agents to provide autonomous, interactive, and crucially accessible mental health support. Existing digital therapies have suffered from low adherence and concerns about their efficiency without continued human support [12,13]. Existing digital therapy formats tend to focus on psychoeducation and a modular style of fixed content and duration that is inflexible for users. Conversational agents hold particular promise compared with other digital mental health interventions as they can provide greater interactivity that emulates therapeutic conversation and provides choice and control over session content and intensity. Research has demonstrated that users respond and connect to conversational agents in social ways, and they can encourage honest disclosure [14,15]. They also have potential for greater scalability compared with other therapy modalities such as human therapists, *Wizard of Oz* programs (where a therapist responds via a computer), or digital interventions that require ongoing support from a clinician to produce favorable outcomes.

The application of conversational agents in mental health is varied and includes diagnostic tools, symptom monitoring, and treatment or intervention [16]. Existing systematic and scoping reviews of conversational agent interventions in the mental health field have focused on a subset of conversational agents with a visual character (embodied) [8-10] or are now outdated [7]. As far as we are aware, this is the first comprehensive systematic review of conversational agents in the treatment of mental health problems.

### Objectives

We conducted a systematic review and synthesis of conversational agents in the treatment of mental health problems. Conversational agents are diverse in design [1] and include, for example, chatbots (eg, casual conversation delivered verbally or through text), embodied conversational agents (ECAs; a virtual visual character that simulates human style, face-to-face conversation with gestures, and nonverbal behavior), conversational agents with a physical presence (eg, robots), and conversational agents within virtual reality (VR). For this systematic review, studies that included an automated conversational agent that simulated a 2-way, real-time conversation, with text or verbal based input (either fixed response options or free text) and an independent (not supported by a human) stand-alone system were included. Studies that used *Wizard of Oz* methods, where a person or therapist responds through the computer or programs that required the ongoing support from a therapist or similar, were excluded. We followed the Preferred Reporting Items for Systematic Review and Meta-Analysis Protocols guidelines [17,18]. The protocol was registered prospectively at PROSPERO (registration number: CRD42018106652).

## Methods

### Literature Search

A systematic search of the literature was performed in September 2018 and updated in January 2019 using MEDLINE (1946 to August week 5, 2018), EMBASE (1974 to September 2018), PsycINFO (1806 to September 2018), Web of Science (1900 to September 2018), and the Cochrane library (All to September 2018). The search was not restricted by publication year or language. Overall, 3 categories of search terms were included: (1) relational agent, (2) mental health, and (3) intervention. The Boolean operator AND was used to bring together separate categories and OR was used to combine terms within categories. Keywords were collated from the existing literature, academics in the field of conversational agents, and the Diagnostic and Statistical Manual of Mental Disorders, Fifth Edition [19]. The search strategy included keyword truncations and mappings to subject heading (medical subject heading) that were adapted appropriately for each database. The reference lists of all included studies were handsearched to identify all relevant references. Gray literature, including conference abstracts or proceedings, and dissertations or theses identified through the database searches were also included for screening.

### Eligibility Criteria

Studies were included if they reported on a conversational agent intervention for mental health; the agent was autonomous and could be used independently without support from a human; they simulated conversation; they relied on a turn-taking process with the user; and they reported on a mental health outcome. Review papers were included if all studies that were included met the inclusion criteria for this review. Studies were excluded if the output from the conversational agent was solely predetermined, for example, psychoeducation and not generated in response to user input; they used asynchronous communication, for example, email; they relied on a human user to generate responses (eg, *Wizard of Oz* methods); they required support from a person to operate, for example, a therapist or similar; they were limited to adherence to medication or physical health behaviors, for example, smoking cessation; they focused solely on the technical function, development or programming of the agent; and they lacked sufficient detail to determine eligibility (eg, short conference abstracts). Studies not written in English were translated as required. The review included a diverse range of study designs such as randomized controlled trials (RCTs), quasi-experimental designs, feasibility studies, and mixed method studies.

### Screening

**Figure 1:**
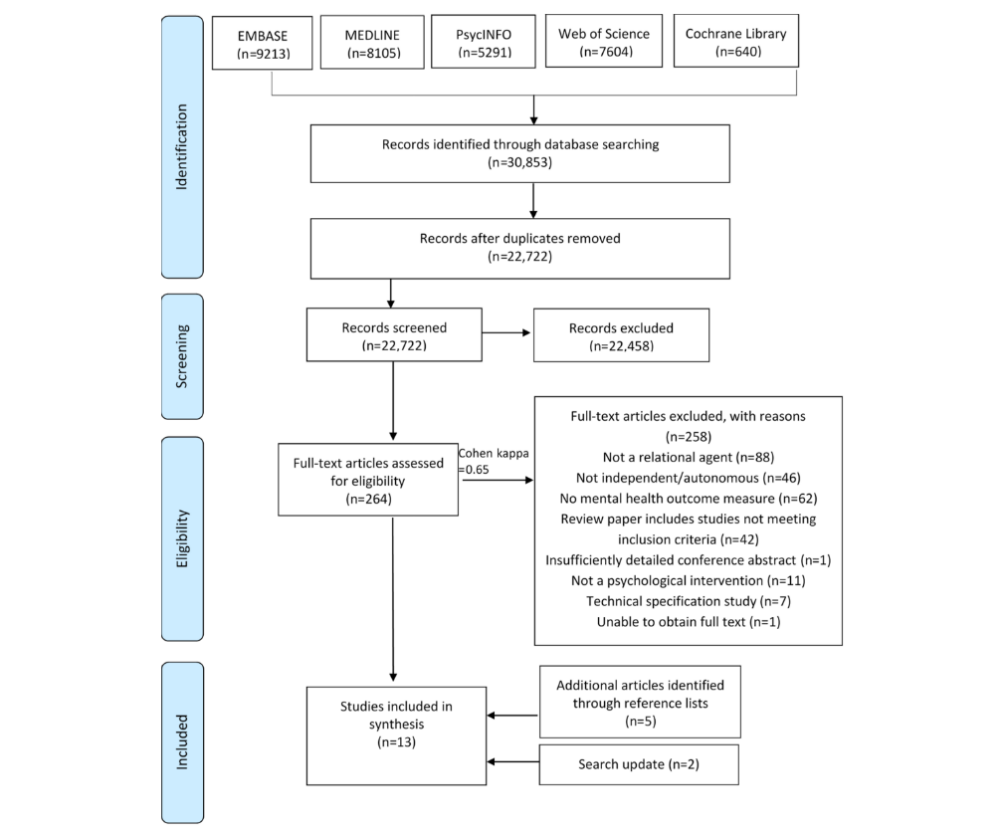
Flow diagram of included studies. Search updates were conducted until January 2019, with 2 new papers being identified.

Studies identified through the database searches were exported to reference management software (Mendeley), and duplicates were deleted. Study selection was conducted by the first author (HG). Screening procedures were piloted before beginning the screening process. Abstracts and titles were initially screened, and articles not meeting the inclusion and exclusion criteria were removed. The first author (HG) then screened full texts and selected the articles for inclusion. Any lack of clarity over the eligibly of the studies was resolved through a discussion with a second author (WM). A random, 9.8% (26/264) sample of studies identified for full-text screening were also independently screened by a second reviewer. Cohen kappa was used to measure interrater agreement. Finally, reference lists of all included papers were screened for additional studies and the inclusion and exclusion criteria applied. See Figure 1 for a detailed breakdown of the flow of the included studies.

### Data Extraction

Data from the included studies were extracted into a prespecified form, which included author, year of publication, study design, mental health domain, conversational agent name and description (including embodiment, access, theoretical approach, and input and output style), number and characteristics of participants (including age, gender, presence, and type of diagnosis or psychological problem), intervention description (including length and structure of intervention), control group description (if applicable), mental health outcome measures, user experience measures, attrition, and primary findings (primary mental health outcome and user experiences). Owing to the diversity in study designs, outcomes measured, intervention modalities, and durations and the varied use of active and inactive control groups, a meta-analysis would not have led to meaningful conclusions and was thus not undertaken. Instead, extracted data were narratively synthesized in line with guidance on the conduct of narrative synthesis in systematic reviews [20].

### Risk-of-Bias Assessment

Risk-of-bias assessment of each study was conducted to ascertain the validity and reliability of the methods and findings to inform the narrative synthesis of the studies. The validated 16-item quality assessment tool for studies with diverse designs (QATSDD [21]) was deemed appropriate for this review to assess study quality as it includes quantitative (14 items), qualitative (14 items), and mixed-methods (16 items) items. Each of the 16 items is rated from 0 (not at all) to 3 (complete). Specifically, the tool assesses the clarity of the theoretical framework, study aims, study settings, the representativeness of the sample, rationale for data collection procedure, the appropriateness and reliability of data analysis, and the study’s strengths and limitations. For each included study, the scores for each item were summed and a percentage of the total possible score was calculated. If a study did not provide enough details to rate an item, the item was scored 0. The quality of each included study was assessed by the first author (HG).

## Results

### Study Selection

The search identified 30,853 articles (see Figure 1) using the predefined search strategy outlined above. Duplicates were removed (8131), and articles not meeting the inclusion and exclusion criteria based on the title and abstract (22,388) were excluded. Handsearching through references resulted in an additional 5 studies being eligible for inclusion. The search was updated in January 2019, and 2 additional eligible studies were identified and included. Lack of clarity over the eligibility of articles (n=13) was resolved through a discussion with the second author (WM). Owing to the large number of articles identified from the initial search and limited researcher resource, interrater reliability was not assessed at the title and abstract stage. However, interrater reliability was assessed at full-text eligibility stage. A random sample of 26 studies (10% of the 264 studies identified for full-text screening) were independently screened by a second reviewer. The percentage agreement between first author (HG) and the independent rater was 96% (25/26 in agreement). Cohen kappa was 0.65, indicating substantial interrater agreement. Any differences in ratings were discussed, and an agreement was reached. A total of 13 articles were included in the review evaluating 11 different conversational agents.

### Risk of Bias

The methodological quality of the included studies varied (see Table 1). Using the QATSDD [21] assessment tool, methodological quality ranged from the lowest score of 35% [22] to the highest score of 88% [23]. The average quality score was 59%. All of the included studies with percentage scores above 70% were RCTs [23-26].

All included studies received the maximum score of 3 for the criterion *statement of aims or objectives in main body of report*. All included studies scored a 2 or 3 for *fit between research question and method of analysis* and *fit between stated research question and method of data collection*. Most studies provided adequate *descriptions of procedure for data collection* and *detailed recruitment data*. Most studies (n=10) provided discussions of the key strengths and limitations of the study (scoring 2), and 3 studies gave thorough, complete discussions of strengths and limitations, obtaining a maximum score of 3. The lowest average scores were found for *representative sample of target group of a reasonable size*, *good justification for analytic method selected*, *assessment of reliability of analytic process (qualitative only)*, and *evidence of user involvement in design*. See Table 1 for mean scores on each criterion across studies.

**Table 1:** List of criteria used to assess methodological quality and average score across studies.

Item	Criteria	Mean^a^
1	Explicit theoretical framework	1.5
2	Statement of aims/objectives in main body of report	3.0
3	Clear description of research setting	2.1
4	Evidence of sample size considered in terms of analysis	1.5
5	Representative sample of target group of a reasonable size	1.3
6	Description of procedure for data collection	2.2
7	Rationale for choice of data collection tool(s)	1.8
8	Detailed recruitment data	2.2
9	Statistical assessment of reliability and validity of measurement tool(s) (quantitative only)	1.5
10	Fit between stated research question and method of data collection (quantitative only)	2.5
11	Fit between stated research question and format and content of data collection tool, for example, interview schedule (qualitative only)	1.9
12	Fit between research question and method of analysis (quantitative only)	2.5
13	Good justification for analytic method selected	1.2
14	Assessment of reliability of analytic process (qualitative only)	0
15	Evidence of user involvement in design	0.4
16	Strengths and limitations critically discussed	2.2

^a^Scores can range from 0 (not at all) to 3 (complete).

### Study Characteristics

The characteristics of the included studies are summarized in Multimedia Appendix 1. The 13 studies identified were conducted between 2013 and 2018 in 4 countries. Among them, 5 studies were conducted in the United Kingdom [23,24,27-29], 6 studies in the United States [22,25,26,30-32], 1 study in Sweden [33], and 1 study in Japan [34]. Across the studies, there was considerable heterogeneity in study design, intervention design, and outcome measures used. The majority of the included studies focused on interventions for common mental health problems, including depression [28-31] and/or anxiety [25,26], specific phobia (heights) [23], loneliness [22], and psychological distress [24,27]. Three studies focused on improving mental well-being [32-34]. A large proportion of studies (n=7) were preliminary and included feasibility [30], pilot RCTs [27,28,31-33], or nonrandomized trials [34]. In addition, 2 studies used quasi-experimental designs [22,29], and 4 studies were full-scale RCTs [23-26].

Most studies (n=8) used mixed methods [22,25,26,28-30,32,33], and the majority (n=9) of them reported on both mental health outcomes and user experiences [22,23,25,26,28-30,32,33].

Over half of the included studies (n=7) used specifically designed control groups, including screen or online psychoeducation [25,26,30,31], paper and CD-/MPEG-1 standard (MP3)-based psychoeducation [32] or an active control condition utilizing another conversational agent ELIZA [2,24,27]. Two studies used treatment as usual (TAU), which consisted of treatment for depression with a clinician [28] or corresponded to no treatment [23]. One study used a waitlist control group [33], and 1 study used a nonrandomized control group of participants who had expressed interest in taking part in the study but could not complete the intervention at that time [34]. Finally, 2 quasi-experimental studies did not use a control group [22,29]. However, Ring et al [22] compared groups that used 2 different versions (proactive and passive) of the conversational agent intervention.

### Participants

Only 1 study, a pilot RCT, recruited participants from clinician caseloads or registers [28]. The remaining studies recruited self-selected participants from the community through outpatient clinics [32], universities [24-27,30,31,33], online advertisements [22,33,34], and radio advertisements [23] and by downloading the intervention app through the app store [29].

The included studies reported results from a total of 1200 participants. Study sample sizes ranged from 14 [22] to 454 [34]. Study participants ranged between 16 and 75 years old, and gender prevalence was 70.3% (692/985) female from studies that reported this data (12/13). One study with 129 participants [29] did not collect data on age or gender, and 1 study recruited only women [32]. Participants varied widely in severity of psychological distress from minimal psychological symptoms [22] to formal clinical diagnoses such as major depressive disorder [28] and acrophobia [23]. In addition, 5 of the 13 included studies recruited participants who self-reported symptoms of psychological distress to varying degrees.

### Conversational Agent Interventions

Overall, 6 of the conversational agents were embodied (7 studies) [22,23,28,30-32,34]. Conversational agents used different technologies, with 3 conversational agents accessed on an app [25,29,33], 4 online (5 studies) [24,26,27,32,34], 3 using an offline computer program (4 studies) [22,28,30,31], and 1 VR program utilizing a VR headset [23].

The majority (8 out of 11 agents, evaluated in 9 studies) of the conversational agents included took natural language input either written [24-27,29,33,34] or spoken [23,28]. The remaining 3 agents took responses from participants using fixed onscreen response options (4 studies) [22,30-32]. The output mainly consisted of questions or written text (6 out of 11 agents, evaluated in 7 studies) [24-27,29,33,34]. Furthermore, 4 agents used spoken output [22,23,28,32]. In addition, 2 studies (1 conversational agent) [31] did not specify whether the conversational agent output was written or spoken.

The conversational agents provided interventions aimed at reducing symptoms [22-29], increasing well-being [32-34], or improving self-management [30,31]. Across the set of conversational agents, a range of therapeutic orientations were used, including cognitive behavioral therapy [23,25,28,34], method of levels (MOL) [24,27], mindfulness-based stress reduction [32], structured communication enhancement strategy [30,31], and eclectic interventions drawing on a wide variety of approaches [22,26,29,33]. Over half of the conversational agents (7 out of 11) focused on providing psychoeducation and self-management strategies [25,26,28,29,32-34], 1 agent (evaluated in 2 studies) utilized the principles of MOL therapy in a question-and-answer format [24,27], 1 agent offered social companionship [22], and 1 agent (evaluated in 2 studies) facilitated practice of effective communication with human health care professionals around psychological symptoms [30,31].

Conversational agent interventions varied widely in frequency and duration (see Table 2). From short interventions of 1 session (participant-determined length [24] up to 20 min [27]), 3 sessions (unspecified duration [31], 15-20 min each [30]), and 6 sessions (30 min each [23] through to daily usage over 2 weeks [25,26,33], 4 weeks [26,28], or a month [32]). One study only used data from participants who had engaged with the intervention at least every other day (>15 times) over a month [34]. Finally, 1 study installed 1 of 2 versions (*passive,* activated at will, and *proactive,* activated by a motion sensor) of the same conversational agent into participants’ homes for 1 week. One study enabled participants to continue TAU for depression with a clinician alongside the conversational agent intervention [28]. The majority of studies (n=9) set no upper limits on usage during the defined study period [22,24-26,28,29,32-34].

**Table 2:** Intervention engagement.

Study	Total intervention length	Frequency of use, mean (SD) or median (IQR)	Intervention duration (min), mean (SD) or median (IQR)
Freeman et al, 2018 [23]	6 × 30-min sessions over 2 weeks	4.66 (SD 1.27)	124.4 (SD 34.2)
Bird et al, 2018 [24]	1 session	Not applicable	13 (SD NR^a^)
Fulmer et al, 2018 [26]	Unlimited access for 2 weeks or 4 weeks	192 interactions (SD NR)	NR
Fitzpatrick et al, 2017 [25]	Daily intervention for 2 weeks	12.1 (SD 2.23)	NR
Ly et al, 2017 [33]	Daily intervention for 2 weeks	17.71	NR
Gaffney et al, 2014 [27]	1 session	Not applicable	19.23 (SD 0.002)
Inkster et al, 2018 [29]	Unlimited access for 2 weeks	83% (90/108) of high-usage users (at least one use) used the app for more than 4 days	NR
Gardiner et al, 2017 [32]	Unlimited access for 30 days	NR	52 (IQR 101.4)
Pinto et al, 2016 [30]	3 × 15-20-min sessions (baseline, 4 weeks, and 8 weeks)	12 of 25 participants completed all sessions	NR
Burton et al, 2016 [28]	Daily intervention over 4 weeks	10.5 (IQR NR)	134 (IQR NR)
Suganuma et al, 2018 [34]	At least 15 times over 1 month	45% (191/427) completed >15 days of intervention	NR
Pinto et al, 2013 [31]	3 sessions (baseline, 4 weeks, and 8 weeks)	NR	NR
Ring et al, 2015 [22]	Unlimited access over 1 week	15.9 (SD 8.1) interactions	2.3 (SD 0.038) each

^a^NR: not reported.

### Feasibility and Engagement

One study reported low uptake as they aimed to recruit 52 participants but closed the study at 28 participants [28]. Attrition rates between pre- and postmeasures were reported in 11 studies and varied widely from no attrition [23,24,33] to 74.1% (1978/2668) of participants [34]. Reasons reported for dropout included difficulties attending the university to take part because of financial difficulties [30], technical problems [22,27], and mental illness [22]. One study with a high attrition rate (74.1%, 1978/2668) [34] did not report any reasons; however, it should be noted that the majority of the dropouts were from the control condition (1846/2109, 88.3%) compared with 23.6% (132/559) in the intervention condition.

Studies reported differing metrics for engagement, and reporting was inconsistent (see Table 2). Engagement with the conversational agent interventions was highly variable from a short period of interaction in 1 session (eg, a mean of 13 min; [24]) to a median interaction total of 134 min [28] or exchanging a mean of 192 messages during intervention [26]. In the study by Suganuma et al [34], 236 out of 427 (55.2%) of intervention participants did not complete 15 or more days of the intervention and were excluded from the analysis. In addition, 3 people (6%) in the study by Freeman et al [23] found the intervention sessions too difficult and did not complete the intervention. However, 44 out of 49 (90%) participants completed the intervention, with a mean total intervention time of 124 min. One study [31] did not report any measures of engagement.

### Psychological Outcomes

Primary outcome measures were all validated but varied (see Multimedia Appendix 1 for details); therefore, the term psychological distress will be used to facilitate a summary. Of the 13 studies included, 5 controlled studies reported significant posttreatment improvements in psychological distress in the intervention group compared with a no treatment or information control group [23,25,26,31,34]. Significant improvements were observed on measures of depression [25,26,31], psychological distress [34], anxiety [26], fear of heights [23] and positive affect [26,34]. Effects ranged from small (*d*=−0.24 [34]) to very large (*d*=2.0 [23]). In addition, 2 pilot trials with active control groups found significantly higher ratings of problem resolution in the intervention group compared with the control group [24,27].

Furthermore, 4 controlled studies reported no significant posttreatment differences on measures of psychological distress between the intervention and control groups [24,27,32,33] with both intervention and control conditions demonstrating reduced distress [24,27,33] or increased uptake of stress management techniques [32]. Despite significant reductions in depression observed in the intervention group compared with the control group in the intention-to-treat analysis by Fitzpatrick et al [25], no significant posttreatment differences in anxiety were observed between groups.

Finally, the 2 uncontrolled studies included in the review [22,29] and 2 studies that did not test for between-group effects [28,30] reported reductions in depression [28-30] and loneliness [22] postintervention. Generally, greater engagement with the conversational agent resulted in greater reductions in psychological distress [22,26,28,29,33]. Only 3 studies included a follow-up period [23,24,27].

### User Experience Outcomes

Generally, from studies that reported user experience outcomes (n=11), participants reported being satisfied with the conversational agent interventions offered [22,23,25,26,29,30,32]. In addition, 3 studies reported that participants found the conversational agent interventions available and accessible [26,32,33]. Participants reported that they found the agent empathic [26], that they liked the interactivity [30], the agent’s personality [22,25], the agent’s ability to form a relationship [28,33], and the agent’s ability to learn from input [26]. Participants reported that they liked the ability to customize the gender and appearance of ECAs [28] and the option to tailor the session length to their own needs [28]. Participants in the study by Fitzpatrick et al [25] reported that they liked the daily check-ins and information provided. Furthermore, 2 studies reported that participants indicated that they would recommend the conversational agent intervention to other people [18,24] (the proactive version).

The predominant challenges to intervention with a conversational agent included repetitive content [22,25,26,28,29,33], limitations in the agents ability to understand or respond appropriately [22,25,26,29], a shallow or superficial relationship [28,33], the sound and quality of the agents voice [32], and specific intervention tools or content [25,29]. Some participants in the study by Pinto et al [30] reported that they would like more frequent, longer intervention sessions, and greater freedom to tailor content and responses to their needs.

## Discussion

### Principal Findings

The use of conversational agents for treating mental health problems appears to be limited but is growing quickly, with 5 of the included studies published in 2018 alone [23,24,26,29,34]. Furthermore, despite the heterogeneity in evaluation methods, there is an increasing emphasis on fully powered RCTs testing efficacy. Included interventions were generally brief, allowed participants to control the intensity of intervention, and drew from a wide variety of psychological approaches. All included studies reported reduced psychological distress postintervention with a conversational agent. In addition, 5 controlled studies demonstrated significant reductions in psychological distress compared with an information or no treatment control group with small-to-large effects. This provides some support for the utility of conversational agents in treating mild-to-moderate psychological distress in adults [23,25,26,31,34]. However, their broader utility in promoting positive well-being in nonclinical populations appears uncertain [32,33]. Controlled studies with active control conditions (eg, another conversational agent or human psychological therapy) failed to demonstrate superior effects [24,27,28]. However, it is important to highlight that these studies assessed relative rather than absolute treatment efficacy, and thus, we cannot conclude an absolute lack of treatment efficacy [35].

Studies managed to recruit participants through several different methods. Remarkably, the only study that reported difficulties in recruiting participants relied on clinicians to refer patients to the study [28]. Studies that used more flexible recruitment routes such as online adverts [34] and app stores [29] recruited greater numbers of participants. It is possible that clinician apprehension about digital treatment for mental health problems affected recruitment rates. This is supported by research indicating that clinicians are perhaps more reluctant to recommend digital interventions without clinician input or support [36,37]. Our findings illustrate that conversational agents are generally an acceptable format of intervention for participants. Interestingly, participants valued aspects of agents usually seen as unique to therapy with a human, such as empathic responses, *personality*, the ability to build a relationship, and an interactive, conversational approach. This is consistent with research demonstrating that people relate to conversational agents as if they were human despite knowing that they are computer programs [38]. Participants also valued the ability of the agent to learn from their input, perhaps emulating the learning of a human therapist over time. Participants found intervention with conversational agents difficult or frustrating when the agent did not understand, became confused, or was repetitive. This perhaps mirrors expectations around core relationship factors such as feeling understood. Control was also important for participants especially regarding tailoring session length and content to their own needs and engaging with interventions in their own words (eg, free-text rather than fixed response options). The accessibility of the interventions was a key strength for many participants and where accessibility was limited, participants highlighted this and suggested ways to improve accessibility (eg, online access [30]).

### Limitations of Included Studies

The studies described have several limitations. The methodological quality of the included studies varied, and sample sizes were mainly small and self-selected, which reduces the ability to draw firm conclusions about the reliability and validity of the findings. Furthermore, because of short or absent follow-up, conclusions about the sustainability of treatment gains cannot be made. Psychological comorbidity was not assessed in any of the studies despite comorbidities being prevalent in individuals with common mental health problems [39]. Safety was only explicitly evaluated and reported in 1 study [30]. Safety is a vital consideration in mental health interventions that use free-text, natural language input either written [24-27,29,33,34] or spoken [23,28]. Studies have demonstrated that these types of conversational agents are often not able to respond appropriately to risk information such as suicidal ideation [40,41] and have the potential to result in harm. Furthermore, users can expect a level of understanding beyond what is currently technologically possible [41]. Engagement with interventions was not reported consistently and appeared highly variable, and the reasons for this remain unexplored. Furthermore, the impact of the design or features of the conversational agents (eg, embodiment and speech or text based) on engagement or outcomes was not explicitly assessed or compared; therefore, conclusions cannot be drawn as to the most effective or acceptable modality. No studies evaluated therapeutic equivalence or superiority to other treatment modalities such as face-to-face therapy. Finally, a large proportion of agents were eclectic interventions comprising a variety of strategies and psychoeducation drawing on a range of therapeutic orientations [22,26,29,33]. Therefore, it is difficult to ascertain what the *active* ingredients of the interventions are.

### Strengths and Limitations

Owing to the lack of standardized terminology in this area, we conducted a comprehensive search that prioritized sensitivity over specificity. We also reviewed reference lists for additional papers not identified through the database searches. Published abstracts commonly presented in technology conferences were also included as they typically provide enough detail for decisions to be made about inclusion. The review was also registered on PROSPERO before commencing. We also included a broad range of formats for conversational agents, including VR and embodied and/or text and speech input. Cohen kappa showed substantial agreement in full-text screening, and there was a high percentage of agreement overall. This is despite inconsistencies in the reporting of interventions which made the process of eligibility assessment more complicated and reflected the heterogeneity and complexity in the field. Owing to the heterogeneity of the included studies, a meta-analysis was not undertaken. Furthermore, some potentially relevant conversational agents developed for the treatment of mental health problems were excluded from this review because of not reporting a mental health outcome measure (eg, ELIZA [2,42-44]).

### Future Directions

Continued growth in the use of conversational agents in mental health treatment is expected. Considering the findings, several priority areas for further research are apparent. First, addressing technical deficits such as repetition and confusion, which were reported in half of the included studies [22,25,26,28,29,33], may help to overcome barriers to engagement. Increased interdisciplinary working between computer science and mental health may facilitate this and help to drive innovations forward. Given that only 1 included study explicitly reported on safety [30], demonstrating safety will also be key to developing patient and public trust [40]. Furthermore, given the range of differing modalities of conversational agents and lack of direct comparisons between them found in this review, it will be important to compare modalities, for example, embodied or nonembodied or speech or text or offer increased choice to individuals. This would enable further insight into what works and for whom. Our review found that a large proportion of conversational agents use an eclectic mix of psychological interventions with often limited theoretical basis [22]. Only 1 included study reported on the process of psychological change [27] with conversational agent Manage Your Life Online (MYLO). Identifying and demonstrating the key mechanisms of action of conversational agent interventions has the potential to increase treatment efficiency, reduce unnecessary burden on users, and increase transparency. Given the diversity of mental health problems (eg, depression, anxiety, and phobias) appearing potentially amenable to treatment with conversational agent interventions, consideration of transdiagnostic approaches to intervention would further increase applicability and reach (eg, to people with comorbidities or difficulties that do not easily fit into prespecified diagnostic categories). Finally, in line with guidance on research priorities for digital interventions [45], it will be important to demonstrate efficacy and/or superiority compared with alternative conversational agent interventions and other treatment modalities such as face-to-face therapy to develop patient and clinician confidence in this type of intervention.

### Conclusions

This systematic review provides an assessment of conversational agent interventions used for the treatment of mental health problems. On the basis of the current evidence, the efficacy and acceptability of conversational agent interventions appears promising compared with no treatment or information control. However, studies failed to demonstrate superiority when compared with other active, conversational interventions, and their broader utility in promoting well-being in nonclinical populations is unclear.

Therefore, whether conversational agent interventions are an adequate substitute to other therapy modalities remains unclear. Future studies should strive to demonstrate efficacy, equivalence (or superiority), and cost-effectiveness through RCTs with comparisons with other forms of treatment. Studies that can demonstrate exactly how interventions achieve psychological change and for whom will be important in streamlining bloated interventions to increase acceptability. Finally, transdiagnostic approaches to treatment may provide further opportunity to maximize the reach and simplicity of conversational agent interventions.

## Appendix

Multimedia Appendix 1 Characteristics of included studies.

## Abbreviations

ECA: embodied conversational agent
MOL: method of levels
QATSDD: quality assessment tool for studies with diverse designs
RCT: randomized controlled trial
TAU: treatment as usual
VR: virtual reality
