# Supplementary material for: Conversational Agents in the Treatment of Mental Health Problems: Mixed-Method Systematic Review
Source: JMIR Ment Health. 2019 Oct 18;6(10):e14166. doi: 10.2196/14166 (PMC6914342; doi:10.2196/14166)
Supplement: Multimedia Appendix 1 [file mental_v6i10e14166_app1.pdf]

Multimedia Appendix 1. Characteristics of included studies.

Author, year, country; mental health domain; QATSDD[a] score (%)	Conversational agent name and description; intervention approach and description	Study type, methods, and participant characteristics	Primary mental health outcome(s)	Engagement and primary user experience outcome(s)
Freeman et al, 2018, The United Kingdom [23]; acrophobia (88)	*Now I Can Do Heights*; VR[b]; speech input and output; embodied; *CBT*[c]; virtual coach delivers CBT for fear of heights including behavioral experiments, belief ratings, and psychoeducation	Single-blind RCT[d] (2-week intervention with 4-week FU[e]) + panel of participants provided verbal feedback on the intervention; 100 adults with a fear of heights (≥30 on HIQ[f]) self-selected from community; intervention group: n=49, 6 × 30 min sessions 2-3 times per week for 2 weeks; median age 45 years (IQR[g] 30-53); 41% (20/49) female; 96% (47/49) white; mean duration of fear of heights 32.0 years (13.8); 86% (42/49) diagnosis of acrophobia; control group: n=51; TAU[h] (equivalent to no treatment); median age 46 years (IQR 38-53); 63% (32/51) female; 88% (45/51) white; mean	Significantly reduced fear of heights (HIQ) posttreatment; effect size d=2.0, *P*<.001; sustained at FU; 69% (34/49) fell below entry criterion at FU (<30 on HIQ) compared with none of the control group; adjusting for imbalances in gender at baseline between groups did not alter findings	No attrition; 96% (47/49) attended 1+ VR sessions; mean sessions attended 4.66 (SD 1.27); mean session duration 26.8 min (SD 2.7); mean total intervention time 124.43 (34.23); 92% (45/49) completed VR sessions and 4 people did not complete (3 found it too difficult and 1 could not attend further appointments); levels of discomfort (Simulator Sickness Questionnaire) in VR very low; panel comments reported satisfaction with intervention

		duration of fear of heights 28.4 years (15.0); 94% (48/51) diagnosis of acrophobia		
Bird et el, 2018, The United Kingdom [24]; psychological distress (76)	*MYLO*; online; free text input; text output; *MOL*[i]; agent asks questions aimed at helping participant to shift awareness to higher levels to resolve internal conflict and reduce distress	RCT (1 session intervention with 2-week FU); 171 staff and students self-reporting a problem causing psychological distress; mean age 22.8 years (SD 7.19); 81.6% (141/171) female; intervention group: n=85; one online session of participant determined length; mean problem related distress 6.42 (SD 1.92); mean DASS-21[j] total 34.63 (SD 19.22); control group: n=86; 1 online session with conversational agent ELIZA of participant-determined length; mean problem-related distress 6.34 (SD 1.86); mean DASS-21 total 30.26 (SD 19.69)	No significant differences between intervention and control conditions on self-reported distress, effect size d=−0.14 (*P*=.27), or DASS-21, effect size d=0.18 (*P*=.16); significantly reduced self-reported distress and DASS-21 scores over time in both groups, *P*<.001; intervention rated as significantly more helpful than control, *P*=.001; intervention resulted in significantly higher problem resolution postintervention compared with control, *P*<.001	No attrition between pre and post intervention. FU optional, 60.8% (104/171) attrition; mean intervention session duration 13 min; mean control session duration 5 min; intervention rated as significantly more helpful than control at post intervention and FU, *P*=.001
Fulmer et al, 2018, The United States [26];	*Tess*; online; free-text or fixed response option input including emojis; text output;	RCT (2 week or 4-week intervention) + user satisfaction survey; mixed methods; 75 (74	Significantly reduced depression symptoms (PHQ-9) in intervention group 1 compared with control,	1% (1/75) attrition (control group); intervention groups exchanged 14,238 messages in

depression and anxiety (75)	*Eclectic*; guided activities based on self-reported mood. Uses CBT, mindfulness-based therapy, emotionally focused therapy, ACT[k], MI[l], self-compassion therapy, and interpersonal psychotherapy approaches. Tess learns over time which intervention styles participants prefer and decreases or increases content accordingly.	completed) university students from 15 US universities; mean age 22.9 years; 70% (52/74) female; 43% (32/74) white; intervention group: n=50; unlimited access to Tess online via an instant messenger app with daily check-ins for 2 weeks (group 1) or biweekly check-ins for 4 weeks (group 2); group 1 (n=24): mean age 24.1 years (SD 5.4); 71% (17/24) female; mean PHQ-9[m] score 6.67 (SD 4.6); mean GAD-7[n] score 6.71 (SD 4.0); mean positive affect (PANAS[o]) 19.88 (SD 1.4); mean negative affect (PANAS) 13.08 (SD 1.3); group 2 (n=26): mean age 22.19 years (SD 2.8); 73% (19/26) female; mean PHQ-9 score 7.04 (SD 4.9); mean GAD-7 score 7.5 (SD 4.9); mean positive affect (PANAS) 21.31 (SD 1.3); mean negative affect (PANAS) 14.38 (SD 1.3); control group:	effect size d=0.68, *P*=.03; significantly reduced anxiety symptoms (GAD-7) in intervention group (G1: *P*=.045; G2: *P*=.02) compared with control; significantly reduced PANAS scores in intervention group 1 compared with control, *P*=.03.	total; mean messages exchanged 192; group 1 exchanged a mean of 283 messages (SD 147.6); group 2 exchanged a mean of 286 (104.6); 86% (43/50) of participants were satisfied with intervention compared with 60% (14/24) of control; the best things about intervention were accessibility, empathy, and learning; the worst things about intervention were limitations in natural conversation, being unable to understand certain responses, and getting confused by answers

		n=24; information control. Online link to National Institute of Mental Health eBook on depression; mean age 22.5 years (SD 4.0); 67% (16/24) female; mean PHQ-9 score 8.17 (SD 4.2); mean GAD-7 score 9.46 (SD 3.9); mean positive affect (PANAS) 22.13 (SD 1.4); mean negative affect (PANAS) 15.75 (SD 1.3)		
Fitzpatrick et al, 2017, The United States [25]; depression and anxiety (71)	*Woebot*; App; free-text or fixed response options input including emojis; text output; *CBT*; *onboarding* (socialization); guided exercises and psychoeducation; general questions about context and mood, for example, "How are you feeling"; links to CBT videos; a "word game" relating to cognitive distortions, psychoeducation, goal setting, regular check-in,	RCT (2-week intervention) + free-text feedback questionnaire; mixed methods; 70 university students with self-reported symptoms of anxiety and/or depression; mean age 22.2 (SD 2.33); 67% (47/70) female; 79% (46/58) white; 46% (32/69) moderately or severely depressed; 74% (52/70) severely anxious; intervention group: n=34; brief, daily CBT informed intervention; mean age 22.58 (SD 2.38); 79% (27/34) female; 82%	Significantly reduced depression symptoms (PHQ-9), effect size d=0.44 (intention-to-treat), *P*=.04 compared with control; study completers (both groups) experienced a significant reduction in anxiety symptoms (GAD-7), effect size d=0.37, *P*=.004; no change observed in affect (PANAS).	17% (12/70) attrition; 31% (11/36) control; 9% (3/34) intervention; mean frequency of interaction 12.1 times (SD 2.23); significantly higher satisfaction with intervention overall (*P*≤.001) and with content (*P*=.021) compared with control; participants liked the daily check-ins (n=9); intervention's "personality" (n=7) and information provided (n=12); participants reported intervention had difficulty understanding some responses (n=10); some technical

	daily or bi-daily usage prompts, weekly mood charts	(22/34) Caucasian ; mean PHQ-9 score 14.30 (SD 6.65); mean GAD-7 score 18.05 (SD 5.89); mean positive affect (PANAS) 25.54 (SD 9.58); mean negative affect (PANAS) 24.87 (SD 8.13); control group: n=36, information control. Online eBook entitled "Depression in college students"; mean age 21.83 (SD 2.24); 55% (20/36) female; 75% Caucasian; mean PHQ-9 score 13.25 (SD 5.17); mean GAD-7 score 19.02 (SD 4.27); mean positive affect (PANAS) 26.19 (SD 8.37); mean negative affect (PANAS) 28.74 (SD 8.92)		problems (n=8); problems with content and repetitiveness (n=2)
Ly et al, 2017, Sweden [33]; well-being (65)	*Shim*; App; free text or fixed response option input; text output; *Eclectic*; tailored questions and psychoeducation; guided exercises and activities using positive psychology	Pilot RCT (2-week intervention) + semistructured interview (20-30 min) focused on positive and negative aspects of intervention; mixed methods; 28 adults; self-selected community sample (university, online and social	No significant difference between groups at post intervention on the FS, effect size d=0.01 *P*=.20, PSS-10 effect size d=−0.96, *P*=.28, or SWLS effect size d=0.17, *P*=.28 (intention-to-treat); for intervention completers (n=13, active at least	No attrition; 1 person in intervention did not complete 14 daily reflections or was inactive for 7 or more days in a row; 78.6% (11/14) participants active 50% or more days); mean frequency of app opening 17.71 (SD 15.7);

	approaches (expressing gratitude, practicing kindness, replaying positive experiences, engaging in enjoyed activities) and third wave CBT strategies (present moment awareness, valued directions; committed actions; empathic responses); daily check-ins; weekly summaries.	media); not receiving psychological therapy or medication; mean age 26.2 (SD 7.2); 54% (15/28) female; 64% (18/28) students; intervention group: n=14; daily intervention; mean age 21.1 (SD 8.8); 50% (7/14) female; mean FS[p] score 44.43 (SD 5.9); mean PSS-10[q] Score 15.36 (SD 5.2); mean SWLS[r] score 25.5 (SD 5.2); control group: n=14; wait-list control group; mean age 25.4 (5.3); 57% (8/14) female; mean FS score 46.14 (SD 4.7); mean PSS-10 score 16.86 (SD 5.0); mean SWLS score 25.86 (SD 3.9)	25% of the days and not inactive for ≥7 days), a significant difference between groups post intervention on the FS effect size d=0.14, *P*=.032 and PSS-10 effect size d=1.06, *P*=.048. No significant difference in SWLS, effect size d=0.37, *P*=0.10.	mean active days 8.21 (SD 3); qualitative feedback (n=9), themes: Negative—repetitive content; shallow relationship; lack of notifications. Positive—learning; available; accessible; perception of app as real person; able to form relationship
Gaffney et al, 2014, The United Kingdom [27]; psychological distress (62)	*MYLO*; Online; free text input; text output; *MOL*; agent asks questions aimed at helping participant to shift awareness to higher levels to resolve internal conflict and reduce distress	Pilot RCT (2-week intervention with 2-week FU) + therapy process analysis; 48 university students self-reporting problem related psychological distress (website and posters); mean age 21.4 (SD 3.1); 79% (38/48) female;	No significant differences between intervention and control condition on self-reported distress, effect size d=−0.60 (*P*=.13) or DASS-21, effect size d=0.17 (*P*=.36); significantly reduced distress (self-reported and DASS-21) in both	12.5% (6/48) attrition (4 excluded from analysis due to server malfunction; 1 excluded due to incomplete measures; 1 lost to follow-up) mean usage intervention 19.23 (SD 0.002); significantly higher ratings of

		intervention: n=26; one session (up to 20 min); 68% (18/26) female; mean distress 6.77 (SD 1.85); mean DASS-21 score 36.73 (SD 24.95); control: n=22; one session (up to 20 min) with conversational agent ELIZA; 90% (20/22) female; mean distress 7.10 (SD 1.41); mean DASS-21 score 30.80 (SD 23.08)	groups at post intervention *P*<.01 and sustained (DASS-21) *P*=.05 or significantly improved (self-reported distress) at FU *P*<.01; problem resolution (self-reported) significantly higher for intervention group postintervention, *P*<.05	helpfulness (self-reported) post intervention for intervention group *P*<.05. Therapy process analysis: greater higher-level awareness of problem significantly predicted greater problem resolution *P*=.01
Inkster et al, 2018, The United Kingdom [29]; depression (56)	*Wysa*; App; free text or fixed response options input; text output; *Eclectic*; inbuilt questionnaires for example, PHQ-9 to match symptoms to support; questions, guided exercises and psychoeducation utilizing CBT, DBT[s], MI, PBS[t], behavioral reinforcement, mindfulness, guided micro actions and tools to build emotional resilience	Quasi-experimental (2-week intervention)+ in-app feedback; mixed methods; 129 individuals with symptoms of depression (PHQ-2 score ≥6); global sample whom downloaded app voluntarily from AppStore; diverse time zones 48.1% (62/129) the United States; 26.4% (34/129) Europe; 18.6% (24/129) Asia; intervention: n=129; stratified; high usage, n=108: at least one use between pre and post; mean PHQ-9 score 18.92 (SD NR[u]) low	Significantly reduced depression (PHQ-9) for both high and low usage groups (authors acknowledge may be due to regression to mean); high usage group experienced significantly greater improvement in depression (PHQ-9) compared with low users CL=0.63, *P*=.03 roughly equivalent to d=0.47	83% (90/108) of high usage users used app for more than 4 days; 59.7% (77/129) completed at least one wellness tool; in-app feedback: 92 users provided 282 feedback responses; 67.7% (191/282) rated app experience favorable; found app and tools helpful; conversation helped to feel better; 32% (91/282) rated app less favorable; tools not helpful; did not use the tools; app not understanding or repeating; app self-focused; conversations

		usage, n=21: no usage between pre and post measures; mean PHQ-9 score 19.86 (SD NR)		"bothered" the user; users who reported it was "hard to cope" rated app significantly more favorably than users who reported "not hard or slightly hard to cope"; there were 1.6% (128/8075) instances of "objection" from the 129 users
Gardiner et al, 2017, The United States [32]; well-being (stress management) (54)	*Gabby*; online; fixed response options input; speech output; embodied; *MBSR*[v]; guided exercises and psychoeducation (MBSR), for example, being present in the moment; responding and not reacting to stress; awareness of breath meditations; body scan; mindful eating; mindful yoga; progressive muscle relation; guided imagery.	Pilot RCT (feasibility; 30-day intervention); mixed methods; 61 women self-referred from outpatient clinics and BioMed Central online newsletter; mean age 35 (SD 8.4); 51% (31/61) white; intervention: n= 31; daily (no time limit); mid-intervention reminder T/C[y] or email; 48% (15/31) white; mean age 33 (SD 8.1); mean PHQ-9 score 7 (SD 4.7); mean SF-12 MCS[x] score 61 (SD 11.6); mean PSS score 17 (SD 3.7); mean frequency stress management techniques used in past week 1 (SD 2); control: n=30;	Number of stress management techniques used increased in both groups post intervention (mean 1 to 4 intervention group, mean 2 to 3 for control). No significant difference between groups despite a trend favoring intervention group. No significant differences between groups post intervention on depression (PHQ-9; *P*=.82), usual activities (SF-12 MCS; *P*=.46) or stress (PSS; *P*=.07). A significant reduction in alcohol use for stress management in intervention condition *P*=.03	7% attrition (4/61) overall (intervention group 9.7% [3/31]; control group 3.3% [1/30]). Feasible; intervention used median 52 min (IQR 101.4); women favored using intervention compared with control; 70% (19/27) of women used intervention information to manage stress compared with 66% (19/29) of controls. Intervention feedback: Benefits—fast, reliable, credible; Challenges—sound and quality of voice, time commitment and accessibility

		information control, same content as intervention delivered via worksheets and CD or MP3 meditations; mid-intervention reminder T/C or email; 53% (16/30) white; mean age 37 (SD 8.4); mean PHQ-9 score 7 (SD 4.6); mean SF-12 MCS score 59 (SD 9.8); mean PSS score 18 (SD 3.5); mean frequency stress management techniques used in past week 2 (SD 2.6)		
Pinto et al, 2016, The United States [30]; depression (50)	*eSMART-MH*; Computer; embodied; fixed response options input; output method NS[z] *SBAR3*[aa]; interaction with virtual healthcare staff with virtual coach who provides tailored feedback and psychoeducation to facilitate effective communication about depressive symptoms	Feasibility and acceptability analysis of RCT ([31] also included) [3-session intervention over 8 weeks with post measures at 12-weeks]; mixed methods; 60 young adults (28 completed all measures) self-reporting depressive symptoms for at least 2 weeks; mean age 22 (SD 2.5); 67% (NR) female; 67% (NR) African American; 58% (NR) self-reported a formal diagnosis of	No harm, distress, or adverse events; depression (HADS) reduced between pre (mean 8.08, SD 4.74) and post (mean 6.50, SD 4.23) for the intervention group *P*=.140. No between-group analyses reported	46% (28/60) attrition over 12 weeks (reported difficulties traveling to the university as travel costs not covered); 48% (NR) of intervention participants completed all 3 sessions; participants generally liked the intervention (ratings of 4/5 for most items); participants found intervention and avatars acceptable, mean immersion score 68.46 (SD 21.78) comments

		depression or anxiety in past or present; intervention: n=12; 3 sessions (15-20 min) each spaced 4 weeks apart; mean HADS[bb] depression score baseline 8.08 (SD 4.74);control: n=16; attention control, screen-based education on healthy living (each module 15-20 min); mean HADS depression score baseline 8.50 (SD 3.83)		for example, "It felt real, like I was there"; intervention providers (avatar coach and health care practitioner) acceptable; content acceptable; positive aspects of intervention: interactivity, increased preparedness for real-life interactions, suggested changes to intervention: greater freedom to tailor content and response options; counseling option at the end; more frequent, longer sessions; Online access
Burton et al, 2016, The United Kingdom [28]; depression (52)	*Help4Mood*; computer; embodied; speech and fixed text response options input; speech and text output; *CBT*; CBT informed intervention designed to support patients receiving treatment for depression with a clinician; utilizes symptom self-report tools; daily mood; weekly mood (PHQ-9); sleep;	Pilot RCT (4-week intervention) + semistructured interview of experience with intervention; mixed methods; 28 adults with a diagnosis of MDD[cc] and scoring ≥10 on BDI-2[dd] and currently receiving fortnightly treatment with a clinician (TAU); mean BDI-2 score 20.7 (SD 7.7); 64% (18/28) female. Intervention: n=14; TAU + daily use of intervention at home;	Small improvement in BDI-2 scores in both groups, intervention (−5.7) and control (−4.2); regular users of intervention (at least twice a week) obtained greater benefit, median reduction of 8 points on BDI-2 compared with 3 points for casual users (3-7 days per week). A reduction in BDI-2 score of more than 5 points reflects a clinically important difference	21% (n=7; intervention group 14% [n=2]; control group 36% [n=5]); low uptake (aimed to recruit 52 but closed after 28); median number of times used 10.5; median total duration used 134 min; almost all would recommend intervention to others; liked ability to customize gender and appearance of avatar; tailor session length; able to establish

	positive and negative thoughts; behavioral activation; relaxation. Supplemented by accelerometer measurement of physical activity and acoustic analysis of speech	mid-intervention T/C; mean age 35.3 (SD 12.1); 71% (10/14) female; mean BDI-2 score 19.6 (SD 8.1). Control: n=14; TAU (appointments with a clinician); mean age 42 (SD 10.4); 57% (8/14) female; mean BDI-2 score 21.8 (SD 6.8)		relationship; disliked repetition and "coldness" of agent
Suganuma et al, 2018, Japan [34]; well-being (45)	*SABORI*; online; embodied; free text input; text output; *CBT*; Guided behavioral interventions and psychoeducation; questions aimed at self-monitoring mood; feedback and behavioral suggestions based on input	Nonrandomized pilot trial (1-month intervention); 2668 eligible self-selected adults (employees, students, "housewives") responded to online advert; 454 included (completed post intervention and if in intervention group used intervention for 15+ days); 70.0% (318/454) female; intervention: n=191; Access intervention at least every other day (ie, >15 times in total); mean age 38.04 (SD 10.75); 69.1% (132/191) female; WHO-5-J[ee] mean score 15.03 (SD 5.26); K10[ff] mean 23.58 (SD 9.56); BADS-AC[gg]	Significantly improved positive mental health (WHO-5-J) effect size d=0.09, *P*=.02 in intervention group compared with control post intervention; significantly reduced negative mental health (K10) in intervention group compared with control at post intervention, effect size d=−0.24, *P*=.005; significantly increased behavioral activation (BADS-AC), effect size d=0.16, *P*=.01 for the intervention group compared with control at post intervention; no significant differences observed on avoidance orrumination (BADS-AR) between	Overall, 74.1% (1978/2668) did not compete FU measures; 55% (236/427) of intervention participants did not complete 15+ days of intervention and were excluded from analysis; user experiences not assessed

		mean 16.09 (SD 8.36); BADS-AR[hh] mean 18.51 (SD 8.79); control: n=263; no intervention (expressed interested in intervention but could not partake at that time); mean age 38.05 (SD 13.45); 71.1% (187/263) female; WHO-5-J mean score 15.64 (SD 5.53); K10 mean 23.76 (SD 9.97); BADS-AC mean 15.67 (SD 8.27); BADS-AR mean 17.71 (SD 9.36)	groups post intervention, effect size d=−0.05	
Pinto et al, 2013, The United States [31]; depression (40)	*eSMART-MH*; Computer; embodied; fixed response options input; output not specified; *SBAR3*; interaction with virtual healthcare staff with virtual coach who provides tailored feedback and psychoeducation to facilitate effective communication about depressive symptoms	Pilot RCT (3-session intervention over 8 weeks with post intervention measures at 12-weeks); 28 self-selected young adults with self-reported depression symptoms or diagnosis of MDD; mean age 22 (SD 2.2); 82% (NR) nonwhite; 64% (NR) female; 71% (NR) not taking psychotropic medication or psychotherapy; 69% (NR) scored ≥8 on the HADS; intervention:	Significantly reduced depression symptoms (HADS), *P*=.01 in intervention group compared with control group post intervention	Attrition NR; user experiences not assessed

		n=NR; 3 sessions each spaced 4 weeks apart (duration NR)		
Ring et al, 2015, The United States [22]; loneliness (35)	*Tanya*; computer; embodied; fixed response options input; speech output; *Eclectic*; assesses affective state "How are you" and provides empathic feedback; talks about local sports; conducts a brief social chat; motivational dialogue encourages physical activity to combat symptoms of depression. Two versions created: passive (no sensor), which relies on person to activate and Proactive (sensor), which detects when person walks past and attempts to initiate conversation.	Quasi-experimental (1-week intervention, pre and post) + semistructured interview; mixed methods; 14 (12 completed) self-selected (online advert on job recruiting website) older adults living alone with no significant depressive symptoms (scoring <3 on PHQ-2[ii]); mean age 65 (Range 56-75); 79% (11/12) female; intervention: n=12 stratified; proactive, n=7; passive, n=5	Trend for proactive group to have greater reduction in loneliness (UCLA[jj]) compared with passive group, effect size d=0.48, *P*=.13. Reduction in loneliness score was correlated with average time spent interacting with the agent r=0.7, *P*<.05. Participants reported feeling less lonely (*P*<.01), happier (*P*<.01), and more comfortable (*P*<.01) when talking to the proactive agent compared with the passive agent	14% attrition (n=2; 1 because of technical problems and 1 because of mental ill health); both proactive and passive: mean 15.9 (SD 8.1) interactions per week lasting an average of 140 (SD 2.3) seconds each; posttest satisfaction mean 4.4 (SD 2.3) on scale of 1 (very unsatisfied) to 7 (very satisfied); post-test ease of use mean 1.9 (SD 1.5) on scale of 1 (very easy) to 7 (very difficult); thematic analysis of interviews revealed:<br>Participants liked content that induced positive affect through humor; comforting statements; exercise encouragement.<br>Participants disliked irrelevant topics, repetition, limited topics; 67% described agent's "personality. Most (6/7

				participants) would recommend proactive agent to a friend compared with only 2/5 in passive condition

[a]QATSDD: quality assessment tool for studies with diverse designs.

[b]VR: virtual reality.

[c]CBT: cognitive behavioral therapy.

[d]RCT: randomized controlled trial.

[e]FU: follow-up.

[f]HIQ: heights interpretation questionnaire.

[g]IQR: interquartile range.

[h]TAU: treatment as usual.

[i]MOL: method of levels.

[j]DASS-21: Depression, Anxiety, and Stress Scale-21.

[k]ACT: acceptance and commitment therapy.

[l]MI: motivational interviewing.

[m]PHQ-9: patient health questionnaire-9.

[n]GAD-7: generalized anxiety disorder-7.

[o]PANAS: positive and negative affect schedule.

[p]FS: flourishing scale.

[q]PSS-10: perceived stress scale.

[r]SWLS: satisfaction with life scale.

[s]DBT: dialectical behavior therapy.

[t]PBS: positive behavioral support.

[u]NR: not reported.

[v]MBSR: mindfulness-based stress reduction.

[x]SF12 MCS: short form survey mental health composite score.

[y]T/C: telephone call.

[z]NS: not specified.

[aa]SBAR3: structured communication enhancement strategy.

[bb]HADS: hospital anxiety and depression scale.

[cc]MDD: major depressive disorder.

[dd]BDI-2: Beck depression inventory-2.

[ee]WHO-5-J: WHO-Five well-being index Japanese.

[ff]K10: Kessler psychological distress scale.

[gg]BADS-AC: behavioral activation for depression scale, activation.

[hh]BADS-AR: behavioral activation for depression scale, avoidance or rumination.

[ii]PHQ-2: patient health questionnaire-2.

[jj]UCLA: University of California Los Angeles loneliness scale.

References

22. Ring L, Shi L, Totzke K, Bickmore T. Social support agents for older adults: longitudinal affective computing in the home. J multimodal user interfaces 2015 Mar;9(1, SI):79–88. [doi: 10.1007/s12193-014-0157-0]

23. Freeman D, Haselton P, Freeman J, Spanlang B, Kishore S, Albery E, Denne M, Brown P, Slater M, Nickless A. Automated psychological

therapy using immersive virtual reality for treatment of fear of heights: a single-blind, parallel-group, randomised controlled trial. The Lancet Psychiatry 2018;5(8):625–632. PMID:30007519

24. Bird T, Mansell W, Wright J, Gaffney H, Tai S. Manage Your Life Online: A Web-Based Randomized Controlled Trial Evaluating the Effectiveness of a Problem-Solving Intervention in a Student Sample. Behav Cogn Psychother 2018;1–13. PMID:29366432

25. Fitzpatrick KK, Darcy A, Vierhile M. Delivering Cognitive Behavior Therapy to Young Adults With Symptoms of Depression and Anxiety Using a Fully Automated Conversational Agent (Woebot): A Randomized Controlled Trial. JMIR Ment Heal 2017;4(2):e19. PMID:28588005

26. Fulmer R, Joerin A, Gentile B, Lakerink L, Rauws M. Using Psychological Artificial Intelligence (Tess) to Relieve Symptoms of Depression and Anxiety; A Randomized Controlled Trial. 2018;5. PMID:30545815

27. Gaffney H, Mansell W, Edwards R, Wright J. Manage Your Life Online (MYLO): A pilot trial of a conversational computer-based intervention for problem solving in a student sample. Behav Cogn Psychother 2014;42(6):731–746. [doi: http://dx.doi.org/10.1017/S135246581300060X]

28. Burton C, Szentagotai Tatar A, McKinstry B, Matheson C, Matu S, Moldovan R, Macnab M, Farrow E, David D, Pagliari C, Serrano Blanco A, Wolters M. Pilot randomised controlled trial of Help4Mood, an embodied virtual agent-based system to support treatment of depression. J Telemed Telecare 2016;22(6):348–355. PMID:26453910

29. Inkster B, Sarda S, Subramanian V. An Empathy-Driven, Conversational Artificial Intelligence Agent (Wysa) for Digital Mental Well-Being: Real-World Data Evaluation Mixed-Methods Study. JMIR mHealth uHealth 2018;6(11):e12106. PMID:30470676

30. Pinto MD, Greenblatt AM, Hickman RL, Rice HM, Thomas TL, Clochesy JM. Assessing the Critical Parameters of eSMART-MH: A Promising Avatar-Based Digital Therapeutic Intervention to Reduce Depressive Symptoms. Perspect Psychiatr Care 2016;52(3):157–168. PMID:25800698

31. Pinto MD, Hickman RL, Clochesy J, Buchner M. Avatar-based depression self-management technology: Promising approach to improve depressive symptoms among young adults. Appl Nurs Res 2013;26(1):45–48. PMID:23265918

32. Gardiner PM, McCue KD, Negash LM, Cheng T, White LF, Yinusa-Nyahkoon L, Jack TW, Bickmore BW. Engaging women with an embodied conversational agent to deliver mindfulness and lifestyle recommendations: A feasibility randomized control trial. Patient Educ Couns 2017;100(9):1720–1729. [doi: http://dx.doi.org/10.1016/j.pec.2017.04.015]

33. Ly KH, Ly AM, Andersson G. A fully automated conversational agent for promoting mental well-being: A pilot RCT using mixed methods. Internet Interv 2017;10:39–46. [doi: http://dx.doi.org/10.1016/j.invent.2017.10.002]

34. Suganuma S, Sakamoto D, Shimoyama H. An Embodied Conversational Agent for Unguided Internet-Based Cognitive Behavior Therapy in Preventative Mental Health: Feasibility and Acceptability Pilot Trial. JMIR Ment Heal 2018;5(3):e10454. [doi: 10.2196/10454]
